# Influence of Augmented Reality Appliances on Tooth Preparation Designs—An In Vitro Study

**DOI:** 10.3390/jpm14010037

**Published:** 2023-12-28

**Authors:** Cristina Obispo, Teresa Gragera, Giovanni Giovannini, Álvaro Zubizarreta-Macho, Juan Manuel Aragoneses Lamas

**Affiliations:** 1 Department of Medicine and Medical Specialties, Faculty of Health Sciences, Universidad Alcalá de Henares, 28801 Madrid, Spain; cristina.obispo@edu.uah.es; 2 Faculty of Dentistry, Universidad Alfonso X el Sabio, 28801 Madrid, Spain; tgragali@uax.es (T.G.); giovanni@uax.es (G.G.); or jmaragoneses@gmail.es (J.M.A.L.); 3 Department of Surgery, Faculty of Medicine and Dentistry, University of Salamanca, 37008 Salamanca, Spain; 4 Department of Dentistry, Universidad Federico Henríquez y Carvajal, Santo Domingo 11114, Dominican Republic

**Keywords:** dental prostheses, conservative dentistry, tooth preparation, full-coverage restoration, complete-crown, augmented reality

## Abstract

The aim of this work was to analyze and compare the tooth structure removal between a free-hand preparation technique and a computer-aided preparation technique using an augmented reality appliance for complete-crowns preparation designs and “root mean square” (RMS) alignment value. Ten upper teeth representatives of all dental sectors were selected from a generic model library as “Standard Tessellation Language” (STL-1) digital files and 3D-printed in an anatomically based acrylic resin experimental model. Then these were randomly assigned to the following tooth preparation techniques: Group A: free-hand preparation technique (*n* = 5) (FHT) and Group B: computer-aided preparation technique using an augmented reality appliance (*n* = 5) (AR). Experimental models were submitted to a digital impression through an intraoral scan and (STL-2) uploaded into a reverse engineering morphometric software to measure the volumetric reduction in the planned and performed tooth structure (mm^3^) and RMS using the Student’s *t*-test and the Mann–Whitney non-parametric test. Statistically significant differences were observed between the volumetric reduction in the planned and performed tooth structure (mm^3^) of the AR and FHT study groups (*p* = 0.0001). Moreover, statistically significant differences were observed between the RMS of the planned and performed tooth preparations in both the AR and FHT study groups (*p* = 0.0005). The augmented reality appliance provides a more conservative and predictable complete-crowns preparation design than the free-hand preparation technique.

## 1. Introduction

Tooth preparation consists of an irreversible structural reduction technique for the manufacturing of an indirect restoration; however, excessive reduction in the dental tissues may cause pulp tissue damage, and insufficient tooth preparation can influence the outcome of the restoration [1]. Traditionally, conventional tooth preparation techniques are performed by free hand and depend directly on the experience of the operator [2]. Recently, it has been recommended to control the reduction in dental tissue by using silicone guides [3], the depth-gauge technique [4], the occlusal record technique [5,6], and the trial restoration approach, since the conventional tooth preparation technique did not provide accuracy nor repeatability [7]. Therefore, the three-dimensional reduction in dental tissue constitutes a challenge for clinicians, since it does not allow an intraoperative measurement of the tooth preparation, which can lead to irreversible complications [8,9]. Specifically, Hsu YT (2004) reported that an inaccurate tooth preparation may cause the failure of both fixed and removable prostheses. In addition, Hsu YT highlighted the difficulty in quantifying occlusal reduction through direct observation, especially in posterior teeth, as well as the design of the tooth preparation of the lingual surface [5]. Likewise, Aminian et Brunton (2003) suggested that insufficient preparation may lead to an inadequate thickness of the prosthetic restoration and an excessive occlusal reduction may compromise both the retention capability of the restoration and the health status of the pulp tissues [6]. Ram et al. (2015) also reported that an inaccurate tooth preparation can also lead to esthetic comprise and biological complications, including caries, periodontal disease, and endodontic or periapical pathology [4].

Thus, clinicians should be encouraged to faithfully reproduce the contours and profiles of dental anatomy for designing full-coverage dental crowns to prevent treatment failure. Ram et al. (2015) described the failure of a full-coverage dental crown as a non-reparable complication, which requires replacement [4]. Therefore, surgical guides can be digitally designed and fabricated using different three-dimensional (3D) printing techniques [10], including stereolithography, inkjet printing [11], and selective laser melting [12]. Various types of 3D-printed tooth preparation guides have been designed for veneer preparation. Both 3D-printed equal-thickness and nonequal-thickness targeted restorative space (TRS) guides have been introduced to visualize the TRS profile for veneer preparation [11]. Gao et al. introduced auto-stop stereolithography guides for simpler and more accurate veneer preparation [13]. Another study suggested that 3D-printed guides could manage the reduction depths in a more controlled and precise manner [12].

The aim of this work was to analyze and compare the tooth structure removal between a free-hand preparation technique and a computer-aided preparation technique using an augmented reality appliance for complete-crowns preparation designs and root mean square (RMS) alignment value, with a null hypothesis (H_0_) stating that there will be no difference between the free-hand preparation technique and the computer-aided preparation technique using an augmented reality appliance for the tooth structure removal and RMS alignment value.

## 2. Materials and Methods

### 2.1. Study Design

**Figure 1:**
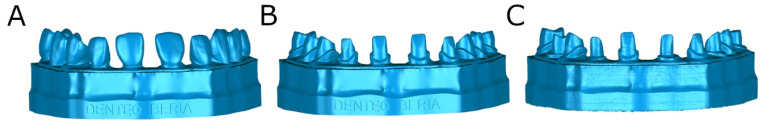
(**A**) Frontal view of the Standard Tessellation Language of the preoperative dental model (STL-1), (**B**) tooth preparations virtually planned (STL-2), and (**C**) the scanned anatomically based acrylic resin experimental model after performing the tooth preparations (STL-3).

Ten upper teeth representatives of all dental sectors were selected from a generic model library (EXOCAD, Darmstadt, Germany) as “Standard Tessellation Language” (STL-1) digital files (Figure 1A) and 3D-printed (Sawbones Europe AB, Malmo, Sweden) in an anatomically based acrylic resin experimental model (Ref.: MED620, Veroglaze, Munich, Germany). Afterwards, the teeth were randomly (Epidat 4.1, Galicia, Spain) assigned to the following tooth preparation techniques: Group A: free-hand preparation technique (*n* = 5) (FHT) and Group B: computer-aided preparation technique using an augmented reality appliance (Hololens1, Redmond, WA, USA) (*n* = 5) (AR). The randomized experimental trial was performed at the Dental Center of Innovation and Advanced Specialties at Alfonso X El Sabio University (Madrid, Spain) between November 2022 and September 2023. Ten complete-crowns preparation designs performed with each preparation technique were included in the study to ensure a power effect of 80.00% for detecting statistically significant differences. The null hypothesis was evaluated using the bilateral Student’s t-test for two independent samples, H₀: μ₁ = μ₂, with a significance level of 5.00%.

### 2.2. Experimental Procedure

Subsequently, ideal tooth preparations for complete crowns were digitally planned using dental planning software (EXOCAD, Darmstadt, Germany) (STL-2) according to the tooth preparation guidelines established by Podhorsky et al. [14] (Figure 1B).

Afterwards, the anatomically based acrylic resin experimental model (Ref.: MED620, Veroglaze, Munich, Germany) was fixed onto an artificial head to simulate the clinical conditions, and the teeth randomly assigned to the FHT study group were prepared according to the tooth preparation designs stated by Podhorsky et al., using a diamond bur surface (ref. 882 314 012, Komet Medical, Lemgo, Germany) fixed to a high-speed rotation device (Tornado LK; Bien Air, Le Noirmont, Switzerland) at 410,000 rpm with profuse irrigation (Figure 1C).

**Figure 2:**
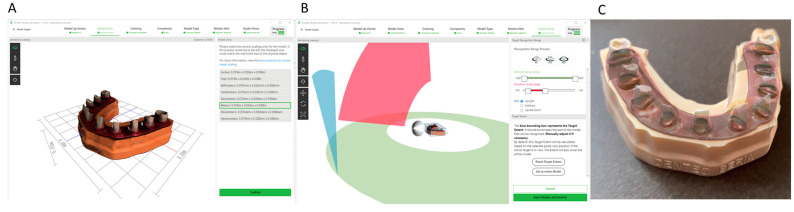
(**A**,**B**) The planning process in augmented reality device software and (**C**) image of the ideal tooth preparations virtually planned over the anatomically based acrylic resin experimental model.

However, the teeth randomly assigned to the AR study group were prepared with a diamond bur surface (ref. 882 314 012, Komet Medical, Lemgo, Germany) fixed to a high-speed rotation device (Tornado LK; Bien Air, Le Noirmont, Switzerland) at 410,000 rpm with profuse irrigation using an augmented reality appliance (Hololens1, Redmond, WA, USA) (Figure 1C). The virtually planned tooth preparation designs at the dental planning software (EXOCAD, Darmstadt, Germany) were uploaded to an augmented reality appliance (Hololens1, Redmond, WA, USA) as an STL digital file, to allow the tooth structure removal procedures in all spatial planes (Figure 2A–C).

Finally, the anatomically based acrylic resin experimental model (Ref.: MED620, Veroglaze, Munich, Germany) was submitted to an intraoral scan (STL-2; True Definition, 3M ESPE ™, Saint Paul, MN, USA) using a 3D in-motion video imaging technology (Figure 1C) (STL-3). The images were captured following the manufacturer’s recommendations by first scanning the occlusal plane, followed by the vestibular and palatal faces.

### 2.3. Alignment Procedure

**Figure 3:**
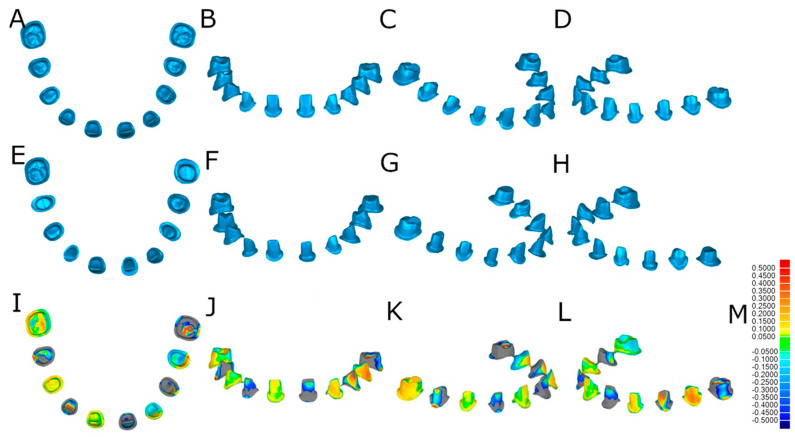
(**A**) Frontal, (**B**) occlusal, (**C**) right lateral, and (**D**) left lateral view of the individually segmented tooth preparations virtually planned (STL−2). (**E**) Frontal, (**F**) occlusal, (**G**) right lateral, and (**H**) left lateral view of the individually segmented tooth after performing the tooth preparations (STL−3). (**I**) Frontal, (**J**) occlusal, (**K**) right lateral, and (**L**) left lateral view of the alignment of the tooth preparations virtually planned individually (STL−2) and the segmented teeth after performing the preparation procedures (STL−3). (**M**) Warm colors represent a volume increase, cold colors represent a volume decrease, and the green represents an accurate alignment.

Once STL-1–3 digital files were imported to reverse engineering morphometric software (3D Geomagic Capture Wrap, 3D Systems^©^, Rock Hill, SC, USA), a full-arch alignment procedure was conducted. The digital files of the anatomically based acrylic resin experimental model (Ref.: MED620, Veroglaze, Munich, Germany) were considered as the reference for alignment, using the best-fit algorithm. Afterwards, the teeth from the STL-1 and STL-2 digital files were segmented (Figure 3A–H) and compared individually in three dimensions using the alignment of the STL digital files (Figure 3I–L).

### 2.4. Measurement Procedure

**Figure 4:**
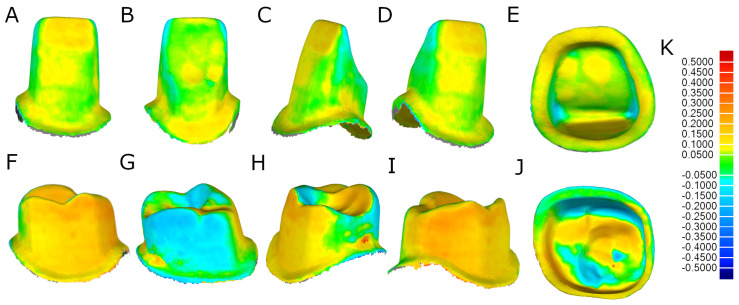
(**A**) Buccal, (**B**) palatal, (**C**) mesial, (**D**) distal, and (**E**) occlusal views of the segmented tooth 2.1 after the alignment of STL−2 and STL−3 using the AR tooth preparation technique. (**F**) Buccal, (**G**) palatal, (**H**) mesial, (**I**) distal, and (**J**) occlusal views of the segmented tooth 2.6 after the alignment of STL−2 and STL−3 using the FHT tooth preparation technique. (**K**) Warm colors represent a volume increase, cold colors represent a volume decrease, and the green represents an accurate alignment.

After the alignment and re-alignment procedures, the following variables were measured: tooth structure removal volume between the STL-2 and the tooth preparations performed with the FHT preparation technique, the tooth structure removal volume between the STL-2 and the tooth preparations performed with the AR preparation technique, the “root mean square” (RMS) between the STL-2 and the tooth preparations performed with the FHT preparation technique, and the RMS between the STL-2 and the tooth preparations performed with the AR preparation technique. The spectrum was set to ±100 μm and the tolerance to ±10 μm (Figure 4K).

### 2.5. Statistical Tests

Statistical analysis of all variables was carried out using SAS 9.4 (SAS Institute Inc., Cary, NC, USA). Descriptive statistics were expressed as means and standard deviations (SD) for quantitative variables. Comparative analysis was performed by comparing the volumetric reduction in the planned and performed tooth structure (mm^3^) and RMS using the Student’s *t*-test and the Mann–Whitney non-parametric test. Normality was tested using the Shapiro–Wilk test. The statistical significance was set at *p* < 0.05.

## 3. Results

**Figure 5:**
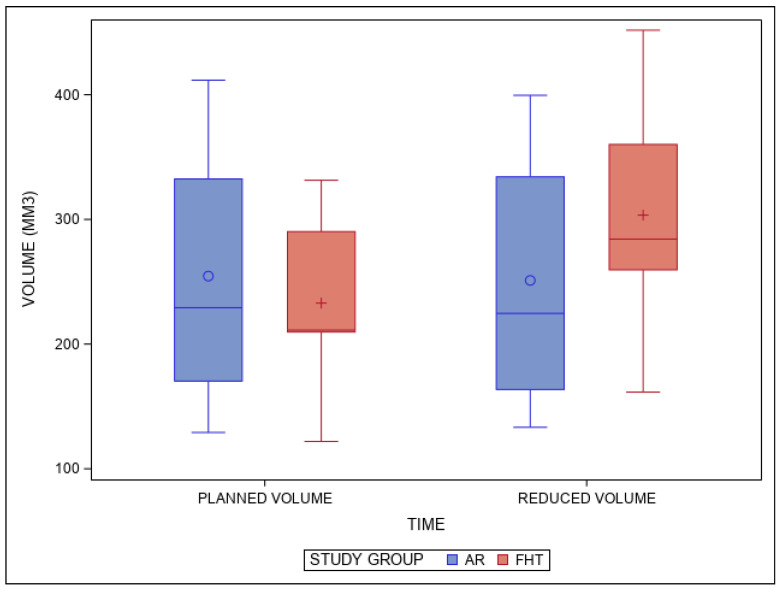
Box plots of the volumetric reduction in the planned and performed tooth structure (mm^3^) of the study groups. The horizontal line in each box represents the median value.

The means and SD values for the volumetric reduction in the planned and performed tooth structure (mm^3^) of the study groups are displayed in Table 1 and Figure 5.

The pairwise comparison revealed statistically significant differences between the volumetric reduction in the planned and performed tooth structure (mm^3^) of the AR and FHT study groups (*p* = 0.0001) (Figure 5).

**Figure 6:**
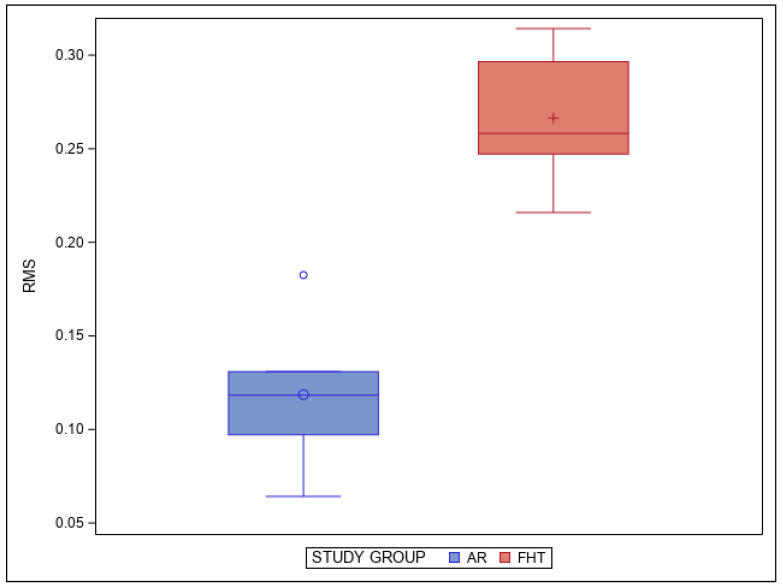
Box plots of the RMS between the STL-2 and the tooth preparations performed with the FHT preparation technique and the RMS between the STL-2 and the tooth preparations performed with the AR preparation technique. The horizontal line in each box represents the median value.

In addition, the means and SD values for RMS between the STL-2 and the tooth preparations performed with the FHT preparation technique and the RMS between the STL-2 and the tooth preparations performed with the AR preparation technique are displayed in Table 2 and Figure 6.

The pairwise comparison revealed statistically significant differences in the RMS between the STL-2 and the tooth preparations performed with the FHT preparation technique and the RMS between the STL-2 and the tooth preparations performed with the AR preparation technique (*p* = 0.0005) (Figure 6).

## 4. Discussion

The results obtained in the present study rejected the null hypothesis (H_0_) that states that there would be no difference between a free-hand preparation technique and a computer-aided preparation technique using an augmented reality appliance for complete-crowns preparation designs and RMS alignment value.

The results reported in the present study showed that the computer-aided preparation technique using an augmented reality appliance provided a more accurate preparation design than the free-hand preparation technique for complete-crowns preparation designs and RMS alignment value.

Traditionally, principles for complete-crowns preparation designs include conservative, retentive, and strong dental reduction, ensuring stability and durability, and an adequate space for the technical reproduction of anatomic contours and profiles with the restoring material. However, inadequate tooth preparations may result in inappropriate labial and palatal contours, leading to compromised esthetics [7]. Additionally, an excessive tooth reduction may weaken the remaining dental structure resulting in pulp complications [11,15]. Therefore, Silva et al. proposed a digital guided tooth preparation design based on digital CAD-CAM technology to achieve predictable and accurate tooth preparation, which showed some advantages compared to the conventional method of tooth preparation for veneers, especially a reduction in chair time for both the one-step and two-step workflows [15]. In addition, Otani et al. used an automated robotic technology for tooth preparation design and reported a mean absolute deviation of 0.133 mm; however, it did not show statistically significant differences (*p* = 0.15) with respect to the free-hand method (mean absolute deviation of 0.112 mm), although the automated robotic technology provided better accuracy for the finish line reproduction [16]. These results differ from ours since the augmented reality technology provided statistically significant differences compared to the free-hand method. This can be due to the fact that Otani et al. designed veneer preparations in maxillary central incisors, and the authors of the present study designed tooth preparations for complete crowns on teeth in all dental sectors, which may increase the differences between both studies. Furthermore, Gao et al. compared the deepness of veneer preparation design between different prosthetic templates and reported statistically significant differences with respect to the free-hand method (*p* < 0.05); however, this study also included veneer preparations in maxillary central incisors [17,18].

In accordance with the above, at present, it has been reported that digitalization in the formation of dental students is used with the aim of increasing the knowledge and to collaborate and communicate among students, teachers, and administrators [19]. Especially in the field of motor skills training, digital software tools can be used to evaluate the manual abilities of potential candidates for the dental curriculum, analyze students’ preclinical preparations, enable self-assessment, and enhance the quality of education. The objective and exact nature of these digital evaluations helps to improve students’ visualization, provides immediate feedback, and enhances instructor evaluation and student self-evaluation and self-correction [19].

Regarding this, Yu et al. comment on the application of microscopic for the dental preparation, comparing to the conventional technique, with silicone rubber guide. The conventional technique provided the lowest precision; although, the silicone rubber guide could not guide the dental preparation because of morphological changes.

Also, in the systematic review of Joda et al., they highlighted the clinical research related to AR/VR technologies in dental medicine and evidenced that were frequently used for skill training [20].

In fact, the development of guiding technologies has improved tooth preparation; nevertheless, these studies were related to the depth of tooth preparation [9].

Likewise, according to the results of Rosella et al., his new preparation technique is more predictable than the conventional technique for no experienced doctors. The reason is that results are less dependent on manual abilities and personal experience. This study has shown benefits in control of depth and direction of tooth tissue removal, as well as a better definition of the tooth finishing line [21].

Bruno Pereira et al. conclude something similar in their study; the veneer-guided prep system allows minimally invasive, digitally guided veneer preparation. In some cases, it enables the application of veneers at the second appointment, alleviating the need for provisional restorations. Some of the system’s limitations can be overcome with the two-step approach, which has the advantage of digitally guided preparation that can potentially increase accuracy, efficiency, and predictability while reducing patients’ chair time. However, the authors highlighted that this computer-aided static navigation technique by therapeutic templates allowed an accurate and minimally invasive tooth preparation design for veneers and enabled the manufacturing of veneers before the tooth preparation [19]. Gao et al. also highlighted the computer-aided static navigation technique for the veneer’s preparation [13], and Luo et al. also reported the high contribution of the computer-aided static navigation technique for the tooth preparation design [22]. Liu et al. used a method to control the depth of the tooth preparation for veneers [23,24]. However, the computer-aided static navigation technique involving stereolithographic templates requires a digital workflow and manufacturing process, which could introduce errors that could influence the outcome of the tooth preparation [25]. In addition, the tooth-supported stereolithographic templates require a high mouth opening capability to insert the therapeutic template and also the high-speed instruments and burs, especially in cases that require tooth preparation of the posterior tooth [26]. Therefore, the authors suggest transferring the preoperative digital planning to the clinical setting without stereolithographic templates. Augmented reality technology has been widely researched in medicine [27] and has provided promising results in dentistry [28], especially in dental implant surgery, where a global deviation of 1.18 mm and angular deviation of 3.96° have been reported. The accuracy of this dental implant placement technology was statistically higher (*p* < 0.001) than the conventional free-hand method, showing a standardized mean difference of 1.01 (95% CI −1.47 to −0.55) [29]. However, the application of augmented reality technology has not yet been analyzed for dental preparation.

However, the study, conducted by Farronato et al., concluded that high-precision cameras with efficient stabilization and the possibility to zoom in a small area are still very expensive and big in size. Also, the landmark of reference is highly influencing the predominant interest in the OMS area; in fact, trials carried out using vascular references, even with high-precision hardware, obtained results, where the outcomes were considered not satisfactory [30].

Furthermore, the use of this technology could simplify digital procedures with direct visualization of virtual information. Timing, although, is more controversial and highly depends on the structure of the system proposed. The timing outcomes are very different from each other; in fact, some are related to the setting and calibration time, while others refer to the duration of the intervention, and the educational studies refer to the time needed to acquire a given skill in dental training and manual dexterity. The positivity in the outcomes and primary endpoints of the considered studies should be taken with caution, since many of the systems described are self-developed by the same institutions as the authors [30].

Additionally, this study has the limitation of being carried out on anatomically based acrylic resin experimental models, which do not provide the operator with the tactile sensation of the hard tissues of the tooth. Furthermore, the teeth are placed in an ideal position in the dental arch, with no rotations, angulations, or dental malposition that could hinder the tooth preparation process. They also do not move, salivate, or close their mouths; therefore, we encourage researchers to continue with this line of work in patients and analyze the outcomes of dental preparations for full-coverage crowns in a clinical environment.

## 5. Conclusions

In conclusion, within the limitations of this study, our results showed that the augmented reality appliance provides a more conservative and predictable complete-crowns preparation design than the free-hand preparation technique.

## Figures and Tables

**Table 1:** Descriptive statistics of the volumetric reduction in the planned and performed tooth structure (mm^3^) of the study groups (mm^3^).

Study Group	Variable	*n*	Mean	SD	Minimum	Maximum
AR	Planned volume	5	254.54 ^a^	116.44	129.10	411.69
	Reduced volume	5	251.03 ^a^	113.12	133.25	339.53
FHT	Planned volume	5	232.87 ^a^	81.13	121.87	331.41
	Reduced volume	5	303.43 ^b^	109.15	161.48	451.76

^a,b^ Different superscripts mean statistically significant differences between groups (*p* < 0.05).

**Table 2:** Descriptive statistics of the RMS between the STL-2 and the tooth preparations performed with the FHT preparation technique and the RMS between the STL-2 and the tooth preparations performed with the AR preparation technique.

Study Group	*n*	Mean	SD	Minimum	Maximum
AR	5	0.12 ^a^	0.04	0.06	0.18
FHT	5	0.27 ^b^	0.04	0.22	0.31

^a,b^ Different superscripts mean statistically significant differences between groups (*p* < 0.05).

## Data Availability

Data are contained within the article.
